# Missed opportunities for digital health data use in healthcare decision-making: A cross-sectional digital health landscape assessment in Homa Bay county, Kenya

**DOI:** 10.1371/journal.pdig.0000870

**Published:** 2025-06-13

**Authors:** Mercy Chepkirui, Stephanie Dellicour, Rosemary Musuva, Isdorah Odero, Benson Omondi, Benard Omondi, Eric Onyango, Hellen Barsosio, Lilian Otiso, Gordon Okomo, Maina Waweru, Maia Lesosky, Tara Tancred, Yussif Alhassan, Simon Kariuki, Feiko terKuile, Miriam Taegtmeyer

**Affiliations:** 1 Malaria Branch, Kenya Medical Research Institute, Center for Global Health Research, Kisumu, Kenya; 2 Department of Clinical Sciences, Liverpool School of Tropical Medicine, Liverpool, United Kingdom; 3 LVCT Health, Nairobi, Kenya; 4 Department of Health, Homa Bay County, Homa Bay, Kenya; 5 GoldInData (GIND), Nairobi, Kenya; 6 National Heart and Lung Institute, Imperial College, London, United Kingdom; 7 Department of International Public Health, Liverpool School of Tropical Medicine, Liverpool, United Kingdom; University of British Columbia, CANADA

## Abstract

The proliferation of digital health systems in Sub-Saharan Africa is driven by the need to improve healthcare access and decision-making. This digitisation has been marked by fragmented implementation, the absence of universal patient identifiers, inadequate system linkages, limited data sharing, and reliance on donor-driven funding. Consequently, the increase in digital health data generation is not matched by similar growth in data use for decision-making, patient-centric care, and research. This study aimed to describe the digital health landscape in Homa Bay County and highlight the strengths and limitations of using digital health data for healthcare decision-making. We used mixed methods. A cross-sectional survey was conducted between June 2022 and October 2023 in 112 healthcare facilities to identify available digital health systems and assess their adoption and utilisation. Thirty-three in-depth interviews were conducted with relevant digital health stakeholders to seek stakeholder perspectives. Our study identified ten different digital health systems, nine of which were in active use. 91% (102/112) of surveyed health facilities had Kenya Electronic Medical Record system deployed for HIV patient management. Eight additional digital systems were available alongside this HIV system, but deployment was fragmented. Challenges to digital systems usage included lack of interoperability, unreliable internet, system downtime, power outages, staff turnover, patient workload, and lack of universal patient identifiers. The study identified multiple systems in use, with the HIV care management system being the most prevalent. The primary challenge hindering effective digital data utilisation is network instability, alongside issues such as the lack of interoperability, disjointed data quality assurance processes, and non-standardised patient identifiers. Recommendations include establishing a routine care data governance framework, implementing universal unique patient identifiers, harmonised data quality practices, advocating for universally compatible digital systems, promoting interoperability, and evaluating the suitability of the existing digital health data for surveillance research and decision-making.

## Author summary

Our paper delves into the evolving realm of digital health adoption and data utilisation in Kenya using Homa Bay County as a case study. Our findings reveal a significant presence of digital health systems, predominantly electronic medical record systems, aimed at improving patient care, data quality, and reporting. Optimum utilisation of these systems is challenged by a lack of interoperability, unreliable internet connectivity, and inadequate data-sharing mechanisms. Notably, we observe a heavy reliance on short-term donor-driven funding, which poses sustainability concerns. Establishing universal patient identifiers, promoting interoperability, and advocating for sustained investment in digital health infrastructure will facilitate better digital data utilisation. By addressing these challenges, we believe Kenya can harness the full potential of digital health technologies to enhance healthcare delivery, decision-making, and research.

## Introduction

The rising use of digital health tools in Sub-Saharan Africa [1–3] is driven by the need to improve data quality, patient care, decision-making, optimising resources, surveillance, research, and healthcare access in line with the universal health coverage agenda [4,5]. Digital health data are predominantly captured in electronic medical records (EMR) systems in healthcare facilities and mobile health applications in community settings. Driven by a public health and patient benefit agenda, digital health data has clear advantages for routine patient care and public health decision-making. For example, accurate, legible records with treatment history enable healthcare providers to identify multimorbidity and potential drug interactions better, offer a cost-effective and efficient means for conducting pharmacoepidemiologic studies, and improve patient data quality and clinical care management [6–9]. The proliferation of EMRs in Sub-Saharan Africa has resulted in the exponential growth of digital health data [10]. However, data utilisation is not yet fully realised in practice, as EMR systems are characterised by fragmented implementation, dual data documentation where both paper-based and EMR systems are used simultaneously, inadequate system linkages and limited data sharing, poor data quality and lack of universal patient identifiers for individual patient data linkages [11,12].

The programmatic approach driving EMR system implementation in Kenya has resulted in multiple disparate EMRs supported by different donors. This EMR ecosystem has been dominated by HIV care EMRs since 2011, evolving into a current nationwide standard EMR for HIV care [2,13,14]. Data for other patient populations are mostly collected through paper-based registers, creating a dual data documentation system. Different project-driven EMR systems hosted through different platforms may operate within the same hospital, though in different departments, such as laboratory and pharmacy, undermining the possibility of a consolidated system to support continuity of care, healthcare decision-making and service improvement [15].

Lack of interoperability and multiple EMR adoption impacts the utility of health data for decision-making and research studies [16]. Interoperability between EMRs has often been hindered by different EMR designs, the type of data collected, the lack of sustainable infrastructure to host integrated platforms and the lack of human resource capacity to develop and utilise integrated databases [13]. In recognition of this challenge, Kenya has developed national guidelines such as Standards and Guidelines for Electronic Medical Record Systems in Kenya, the Kenya Health Information Systems Interoperability Framework, the Kenya National e-Health Strategy, and recently, the Health Sector Unique Identification Framework [14,17–19]. Despite a successful small-scale pilot in Kenya demonstrating how interoperable systems improved data quality and automated reporting for HIV, major obstacles remain in actualising interoperable EMR for healthcare at scale [20]. Interoperability maturity levels of Kenya’s health information systems are low in four major domains: leadership, governance, human resources, and technology [21]. As of May 2023, the country is rated at Phase 2 overall in its digital health journey in the Sub-Saharan region, according to the Global Digital Health Monitor [22]. Kenya has strong areas such as Strategy & Investment (Phase 3) and Infrastructure (Phase 3), with dedicated funding and a division for health informatics within the Ministry of Health. There is also a robust legal framework for data protection and privacy. However, the country lags in areas like Workforce development (Phase 1), where there is a lack of structured digital health training programs.

The absence of universal unique patient identifiers (UPIs) significantly affects data linkage and use in EMR systems. Without UPIs, patient data becomes fragmented across different healthcare facilities, hindering the seamless exchange of medical information between various EMR systems [23]. This fragmentation poses a substantial challenge in sharing patient information and tracking medical records across different healthcare services with distinct EMR systems [23,24]. This is particularly challenging in low-resource settings where paper-based medical record-keeping is prevalent [25]. The absence of UPIs complicates the identification of patients within and across healthcare facilities, leading to difficulties in accurately linking patient records and sharing information between EMR systems [26]. The need for UPIs is further emphasised in the context of EMR implementations, where creating UPIs is essential, especially in regions lacking a standardised identification system [27]. The recent introduction of the Health Sector Unique Patient Identification framework in Kenya [19] for EMR systems is a potential enabler of data linkage, use and sharing if implemented across disparate EMRs.

Poor data quality in EMR systems can hinder effective decision-making in healthcare, leading to compromised patient outcomes, user dissatisfaction, inefficiency, and compromised data reliability. Programmatic EMR has been siloed in low-resource settings to meet different funding agencies’ requirements, leading to inefficiency and poor data quality [28]. Additionally, insufficient staff training to manage data has led to data incompleteness [29–31]. Incomplete and inaccurate data hinders provision of high-quality care and patient monitoring over time [32]. Poor quality data can introduce research biases and population misrepresentation, especially where data missingness is very high [28,33]. The lack of trust stemming from poor data quality creates a vicious cycle that limits data sharing across platforms [34].

This study was conducted in Homa Bay County, Kenya. Homa Bay has a typical Kenya’s healthcare structure [35] with advancing digital health interventions in public health facilities and a committed health care management team. We aimed to describe the digital health landscape and highlight the strengths and limitations of using digital data for healthcare decision-making. Specifically to highlight the frequency of digital health data generation and the level of integration of digital health systems, explore barriers and opportunities for their use in public health decision-making and research, and provide recommendations on actionable steps Kenya’s digital health ecosystem can undertake to achieve effective data utilisations for decision-making.

## Materials and methods

### Study design

We conducted a mixed-methods study, combining a cross-sectional survey with qualitative interviews. A cross-sectional landscape assessment was conducted in Homa Bay County in Kenya between June 2022 and October 2023. We used health facility surveys and in-depth interviews with relevant health system stakeholders to identify which EMRs were operational in healthcare facilities and explore stakeholder perspectives on their utility for public health decision-making. The study was carried out in the same area as two ongoing studies, one focusing on malaria in pregnancy (MiMBa Pregnancy Registry study) [36] and the other on community-facility linkages for antenatal care (C-it Du-it study) [37].

### Study setting

Kenya has 47 Counties and each County is sub-divided into sub-counties. Homa Bay County is located along the shores of Lake Victoria. It comprises 350 healthcare facilities classified as faith-based, Ministry of Health (MOH), non-governmental and private across eight sub-counties [38]. Facilities are grouped into levels: Level 2 (223/350) represents dispensaries and private clinics; Level 3 (101/350) refers to health centres; Level 4 (25/350) refers to sub-county hospitals. Homa Bay has one referral hospital (Level 5) and is one of the counties with the highest density of digital health systems used in healthcare facilities. The rollout of digital health initiatives in public hospitals started with the county referral hospital (Level 5) before moving to Levels 4, 3 and eventually Level 2 facilities.

### Site and participant selection

**Fig 1:**
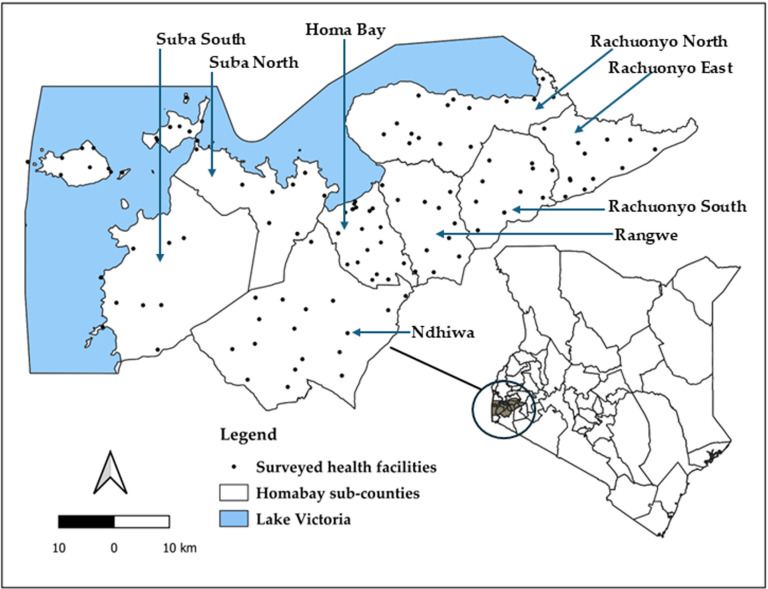
Study area showing spatial coverage of surveyed health care facilities in Homa Bay county. The map was created using QGIS 3.30.1, with basemap shapefiles sourced from https://gadm.org/index.html. Waterbodies were obtained from The World Bank data catalogue (https://datacatalog.worldbank.org/dataset/africa-water-bodies-2015) but are curated from The Regional Centre for Mapping of Resources for Development (RCMRD) Geoportal (http://geoportal.rcmrd.org/layers/servir%3Aafrica_water_bodies#license-more-above).

The landscape assessment covered MOH facilities, private facilities and faith-based facilities across Homa Bay County. The MiMBa Pregnancy Registry study focused on two sub-counties (Suba North and Homa Bay sub-county), encompassing facilities with a consistent supply of antimalarials. MiMBa facilities with an EMR system were included in the landscape assessment. These facilities utilised ScanForm, an AI-based data extraction platform, to convert handwritten registers into digital data, improving data collection efficiency and accuracy [39]. The C-it Du-it study conducted a county-wide situational analysis in 2023, including all health facilities with over 50 births per year. In total, 112/350 sites were included in the landscape assessment (see **Fig 1**), with seven sites overlapping between the two studies.

We employed purposive sampling to select study participants for the qualitative research, ensuring rich and diverse data to explore the research questions [40] effectively. Participants were from Homa Bay County and Kenya’s nationwide leadership and stakeholders in digital health. Participants were chosen based on their experience and/or knowledge of digital health systems, including community health promoters and community health assistants, county and national senior MOH officials, EMR implementing partners, digital health experts, county and facility health record information officers and health facility in-charge. The study included 33 participants, with the sample size determined by theoretical saturation [40].

### Quantitative data

#### Health facility surveys.

We used structured questionnaires for our landscape assessment. Questionnaires were completed with health records information officers, facility in-charges, and data clerks interacting with an EMR daily. Survey variables included facility demographics; EMR adoption and use; EMR adoption challenges; patient identification methods; and data sharing. Survey tool was informed by existing digital health literature in Kenya. The tool was piloted in the MiMBa sites in a first phase of data collection (June–August 2022) and adapted for data collection (February–May 2023) in C-it DU-it sites. One hundred and twelve of the 350 facilities were surveyed in eight sub-counties; 37.5% (42/112) were dispensaries (level 2), 41.7% (46/112) health centres (level 3), 20.5% (23/112) sub-county (level 4) and one county (level 5) – (see **Fig 1**). The surveys were carried out by a data manager (MC), county-appointed health records information officers and trained research assistants.

#### Quantitative data analysis.

Two survey databases were cleaned and then merged using R software version 4.3.1. A descriptive summary of EMR utilisation, patient identification method, data reporting routine, EMR challenges and data quality assurance processes were generated and classified by facility level.

### Qualitative data

#### Sampling and data collection for qualitative research.

Qualitative data were collected between May and September 2023 through in-depth interviews with national, county and facility stakeholders. The interviews were conducted in English or Luo, depending on participant preference. Most interviews were conducted face-to-face in secure locations within facilities, with a few conducted via telephone for participants living outside Homa Bay County. Each interview lasted about an hour, was audio recorded and supplemented with written notes to ensure accuracy. Experienced qualitative researchers, well-versed in national and local health systems, conducted the interviews after training in the study protocol and research ethics. Semi-structured guides for the interview covered various topics, including digital health systems, digital health policy development, data protection, and interoperability and systems linkage.

#### Qualitative analysis.

The interviews were transcribed using a denaturalised approach and checked for accuracy and completeness [41]. Interviews conducted in Luo were simultaneously translated into English during transcription, and all scripts were quality appraised, with the translated transcript checked against the original audio. Data were analysed in Nvivo12 using a framework analysis approach [42,43]. We developed a coding framework based on a sample of transcripts, a topic guide and research objectives, piloted and revised. Each transcript was systematically analysed using the coding framework to identify relevant codes, categories, and themes. Initial analysis of quantitative data facilitated the identification of relevant qualitative data to triangulate emerging quantitative findings, including barriers and opportunities for utilising EMR data. To ensure rigour and trustworthiness, transcripts were independently coded, compared, and discussed among the authors [44]. Finally, a validation workshop involving a sample of the research’s previous participants was conducted in October 2023 to discuss emerging findings from our initial analysis to strengthen our interpretive validity. Feedback from the workshop —which largely reflected changes since data collection—was integrated into our results.

## Ethics statement

This study was approved by local and international ethics committees: The Jaramogi Oginga Odinga Teaching & Referral Hospital Institutional Scientific Ethics Review Committee (ISERC/JOOTRH/676/22) and Liverpool School of Tropical Medicine Research Ethics Committee (LSTM REC 22–069). Additionally, the studies and EMR landscape assessment were granted approval letters from the Homa Bay County, Ministry of Health. Verbal consent was sought from the health facility healthcare workers before the quantitative survey was conducted. No personal data were collected/recorded when conducting EMR landscape assessment. Written informed consent was administered to all C-it-Du-it study participants to participate in the study and to allow for audio recording of the qualitative interviews.

## Results

### EMR coverage

**Fig 2:**
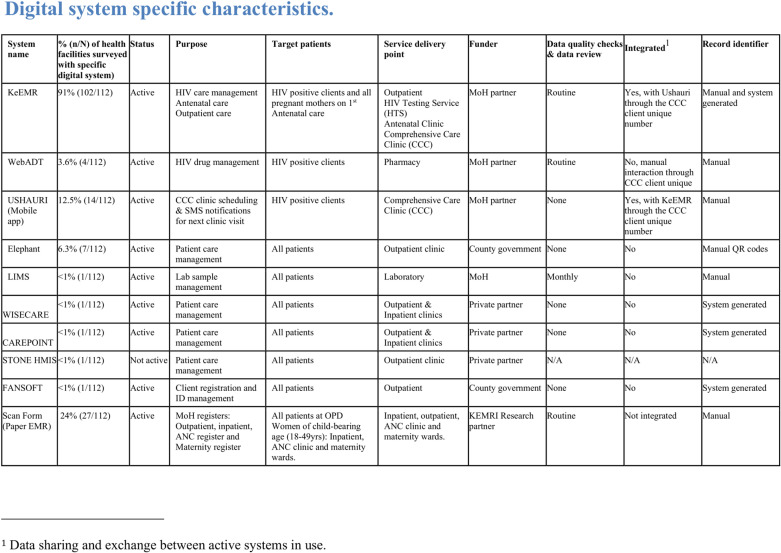
System-specific characteristics. Ten digital systems in Homa Bay are described in their active status, purpose, target patients, sponsor institution, interoperability status, EMR record identification, and data quality review status.

One hundred twelve health facilities were assessed. Our study identified ten different digital health systems, nine of which were in active use. Ninety-three percent (104/112) of the health facilities surveyed had an EMR: 91% (102/112) had Kenya Electronic Medical Record system (KeEMR), which was deployed for HIV patient management and care supported by LVCT Health (an established Kenyan NGO and the main implementing partner for HIV services in the county). Eight additional EMR systems were identified in active use alongside KeEMR: two to supplement HIV care; two for general population care at level 4 hospitals and above; two independent systems developed and funded by vendors for the faith-based sector; and one laboratory information management system unique to the county referral hospital (see **Fig 2**). Two systems were independently deployed in facilities with no KeEMR. However, one was inactive. An AI-based digital data technology, ScanForm, that scans paper-based clinic registers, was used in the facilities supported by the MiMBa project, comprising 24% (27/112) of the facilities. Eight facilities were identified as not having EMR: seven were level 2 hospitals, and one was level 3.

**Fig 3:**
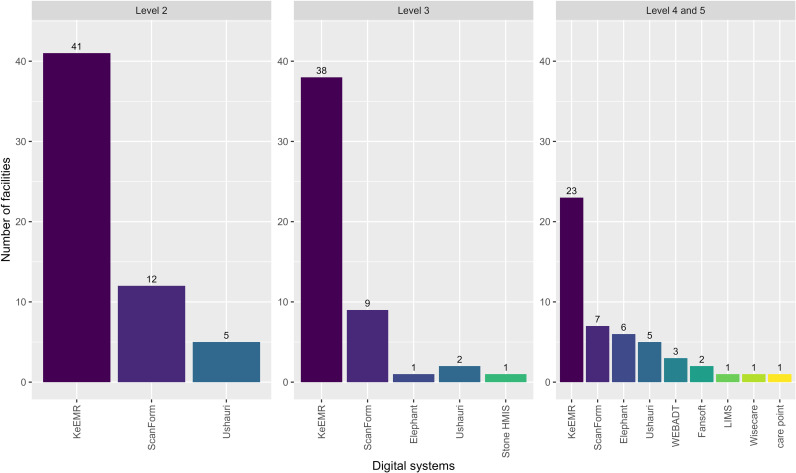
Digital systems in health care facilities in Homa Bay county.

Six of the nine active EMRs were supported by implementing partners and faith-based organisations. The County Ministry of Health (MOH) supported two, and the laboratory information management system was funded by the national MOH (*see* Fig 2). The number of systems available in a facility increased as the size and facility level increased, with the level 5 hospital having seven EMRs (see Fig 3). Ninety eight percent (102/104) of facilities with an EMR had KeEMR denoted in Fig 3.

Data from the qualitative interviews align with the coverage pattern observed in the survey. Most respondents recognised KeEMR as the primary system in widespread use in facilities while reiterating that EMRs were either restricted to HIV programming or heavily skewed towards HIV service provision. Participants raised a concern regarding KeEMR’s limitation to HIV care in comprehensive care clinics. Participants noted data entry into KeEMR was sometimes done retrospectively.

*The digitalisation that is cutting across the county is the KeEMR. We have the KeEMR that is doing well. But mostly the KeEMR is in the comprehensive care clinics … (County-level respondent, KII001)*

Some respondents pointed out EMR’s extensive geographical coverage and contrasted this with its narrow focus on HIV due to the vertical funding model.

*…because it’s a vertical program you realise that there are components like the [maternal and child health] that are not embracing it. We are pushing so that the [maternal and child health] exists in the system.* (County-level respondent, KII004)

A tablet was the most used device at 76% (84/111) and nurses were the leading category of users at 54% (49/91) who entered data into EMRs (see S1 Table).

### Data quality assurance and reporting

Data quality checks and data reviews for 90% (82/91) of EMRs were routinely done by facility department staff and health records information officers in collaboration with EMR implementers' quality assurance teams. While the data review teams varied from one facility to another, privately acquired EMRs did not have a routine schedule and limited access to data for quality monitoring (see Fig 2 and S1 Table). A lack of mentorship and continuous training of facility staff affected the data quality assurance process. Network issues and workers’ strikes introduced gaps in the data, ultimately reducing data completeness.

Participants reported quarterly aggregate data review meetings with the county level, though it was acknowledged these meetings did not always occur as regularly as intended due to a lack of resources. Frequent reporting didn’t match the data quality, as data accuracy was still an issue.

*We are doing very well in our reporting. But the only question is, is it accurate, is it of quality? So that quality thing is still an issue. But we have tried, we have come from far. (County-level respondent, KII001)*

Before data were sent from facilities to sub-counties, they would be reviewed and analysed. This internal process was important from a quality assurance perspective.

*The facility guys will be able to analyse their data at their level and see how it makes sense for the decision-making within the very facility. (County-level respondent, KII003)*

If there were any gaps in completeness, outliers, or if it seemed there may be an error with the data reported, there was follow-up, usually through sub-county and facility health records officers, who may liaise with specific departments to confirm if the data entered was correct. WhatsApp and email were used to communicate between officials to facilitate these feedback conversations.

National-level respondents spoke about the myriad of data quality assurances and measures to enhance quality and reporting. These included data preloading, field restriction, and validation rules; restriction and standardisation of forms where numerical data is needed; random data quality audits to verify the accuracy of collected data; triangulation of collected data with external sources or surveys; implementation of data lifecycle management; and setting up customised dashboards for data analysis and reporting.

*Data quality again is a process, not an event. So, you would need to map out the data life cycle from data collection, data processing, data storage and then of course now analytics and visualisations and at each one of those stages you would have to identify what are the data quality risks or things that would affect the quality of the data and then you address each one of them. (National-level respondent, KII010)*

### Patient identification

EMR unique record identifiers were manually generated in five of the nine EMRs—three EMRs had a system-generated identifier and one used manual and system-generated techniques. EMR record identifier was manually allocated at the first patient interaction in 97% (107/110) of the facilities. One unique identifier was allocated to HIV-positive clients once at a given facility, which was then used in all HIV care systems (WEBADT, Ushauri & KeEMR) within a facility.

Qualitative data highlighted current efforts around the national unique patient identifier framework and the potential for standardisation. The prospect of using the *Huduma* number—a Kenyan government-issued number used to access government services—as a unique idea was seen as “not a bad idea”.

*So maybe things will change one time and…you can just use a phone, and someone can get you wherever you are. And a software is just put there, and you find yourself when you go to a certain facility, if you click like this maybe all your details come out. It is called NUPI: National Unique Patient Identifier…. It is something that is still going on. (County-level respondent, KII001)*

There was no county-specific universal patient identification system. A common challenge cited was individuals being assigned multiple numbers for different purposes, i.e., at the inpatient, outpatient and antenatal clinics and may forget their numbers or the cards on which the unique numbers are recorded. Others talked about the advantages of the universal identifier for individual patient treatment follow-up within and across facilities, as illustrated by this county-level respondent.

*… the advantage of having a unique number is that it gives you the exact number of clients that come to the facility. It reduces duplication of numbers. And when you have one file for one person, you don’t keep on opening file for one person for so many years… Most patients don’t even know what drugs they were given. So, when the patient is on treatment and maybe next week you admit that person and she has forgotten the card. But if it is something they have been given that is unique, you can follow. You can know, ‘this patient last time I gave her this and she is not improving…’ So, changing of treatment is very easy.* (County-level respondent, KII001)

### Interoperability

The nine active digital health systems operating in the county, including the primary national health information system, were not integrated. While the HIV care management EMRs shared data using a common unique client identifier, none of these systems were connected to other EMRs deployed for purposes other than HIV care.

National-level respondents highlighted several challenges to interoperability, namely: the wide range of actors across different systems and limited coordination of these; differences in technology; lack of government leadership and resource allocation to health data systems; an absence of standardisation of processes and technologies for interoperability; and concerns around data quality, as these typical quotes illustrate:

*The other thing is basically the use of standards. It is not all platforms that are built or that are open-sourced or that have been amenable to the use of standards for interoperability. So that has become a barrier and that again perpetuates the custom-based interoperability issues recognised. (National-level respondent, KII008)*

*…we also need to address data quality and data cleaning maybe before we share. And most people don’t have the time to actually conduct data quality audits and data cleaning before the sharing. So, we tend to fear sharing.* (National-level respondent, KII010)

### EMR challenges and benefits

The healthcare workers in the facility survey recognised benefits of EMR in fast record retrieval, secure patient data, ease of records access, automated report generation, saved time and space (compared to paper records storage). They liked how EMR is portable and can be accessed from anywhere.

**Fig 4:**
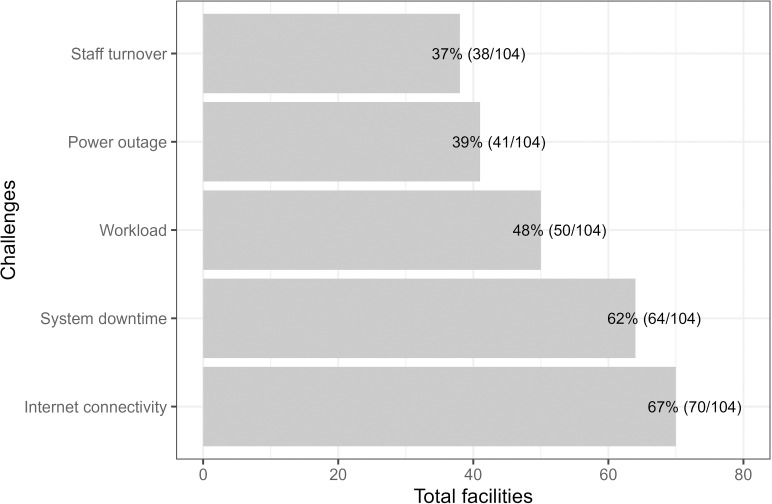
EMR challenges reported in 104 facilities surveyed.

**Fig 4** summarises how EMR use was affected by poor internet connectivity, system downtime and power outages, underscoring the enduring appeal of paper registers. Patient workload and staff turnover were also cited as affecting the utility of EMR. These challenges were echoed in the qualitative interviews, as shown in these typical quotes:

*The other bit that others have reported is the issue with low battery, issue of charging of the machine. And then there are other areas where we have also realised the issue of the training. Maybe the transfer—somebody who was in this facility and of course he transferred to another place. Somebody coming in who has not been using the EMR has challenges. (County-level respondent, KII003)*

*…There are times [the EMR] is very slow and one cannot access it but the [paper] registers are always available unless it is destroyed, or it gets lost. So, that makes it easier to access the information of clients when needed. I think that is what makes it stand out than the EMR that is affected by network, inaccessible, and downturns but for the registers, they are always available.* (Facility respondent - IDI004)

Other challenges reported by the survey were a lack of local user support, workers’ strikes, inadequate data bundles, HRIOs not fully benefiting from the EMR adoption as manual tallies of the reports were still required, and few trained staff at the facility. Ensuring people are adequately trained in EMR, especially in the face of staff turnover, was mentioned in interviews. However, the use of solar panels and leveraging continuous medical education are two approaches that have been put in place to overcome this.

Although the digital systems are currently limited, interview participants were unanimous in remarking it was a good direction of travel. Many felt the county was ready to move towards digital data systems, but resource availability was a major constraint.

*I think the county is ready. The only gap which is there is getting resources to start it off… Even the annual work plan which is being developed now and of course the one which we are going to start using in the next financial year…the bit of digitising the community system and even the facility, I think it is also within the plan. So, we are ready for it and of course, we are mobilising resources so that we can start it off. (County-level respondent, KII003)*

## Discussion

This study aimed to describe the digital health landscape in Homa Bay County and highlight the strengths and limitations of using digital data for healthcare decision-making. We found that 93% (104/112) of rural and urban healthcare facilities had active EMRs encompassing nine different systems, with the largest coverage driven by one EMR for HIV care. The lack of interoperability of existing systems was prominent, exacerbated by many competing systems within the same setting driven by vertical programmes. The latter was particularly true in higher-level facilities, with the county referral hospital managing seven different EMRs. Manual identification of EMR records is predominantly utilised despite the existence of common patient identifiers captured across EMRs that lack standardisation. Participants raised challenges such as frequent power outages, unreliable internet connectivity, lack of mentorship and continuous facility staff training on data management, which affected routine data quality assurance. The manual process of report generation and the lack of resources to host routine data feedback meetings slowed down reporting and data verification for higher-level decision-making.

The positive impact of digitisation on healthcare is undeniable. As we move forward, it is crucial to evaluate the potential benefits and challenges associated with broader deployment of digital health systems in Kenya. EMRs can improve patient care and safety by enhancing adherence to clinical guidelines, reducing medical errors, and improving medication management [45–47]. They also facilitate better coordination and communication among healthcare providers, increase efficiency by reducing redundant tests, and provide valuable data for research and public health monitoring [48–50]. Despite the challenges hindering EMR adoption in Homa Bay, healthcare workers recognise the numerous benefits of electronic medical records. EMRs offer faster record retrieval, enhanced patient data security, easier access to records, automated report generation, and significant time and space savings compared to paper records. The portability of EMRs, allowing access from anywhere, further underscores their value in today’s fast-paced world. From a financial perspective, EMRs offer benefits such as improved charge capture, reduced transcription costs, and support for evidence-based practices, leading to increased physician satisfaction [50]. However, challenges remain, including high upfront and ongoing maintenance costs, potential workflow disruptions, and concerns about privacy and security [51]. Additionally, new technology-related errors, changes in doctor-patient interactions, system downtimes, and interoperability issues can pose obstacles [52]. Some healthcare providers may resist the changes, and there is a risk of overdependence on technology [53]. To fully realise the potential of EMRs, it is essential to address these challenges and develop strategies to ensure a successful implementation and adoption of digital health systems in healthcare settings.

The distortive effect of vertical programming and donor-driven priorities dominates the digital health landscape in Kenya [5,54]. The high coverage of 91% (102/112) we found in Homa Bay was due entirely to KeEMR, a system funded by the HIV vertical programme and used almost exclusively for the care of people living with HIV. The programme-driven EMR results in a siloed and fragmented digital health ecosystem. This fragmentation introduces dual health documentation in settings where paper registers and EMR systems are used simultaneously [12]. The variation in data entry protocols and bespoke implementations of EMR systems can lead to widely varying data quality across institutions [55]. We found routine data quality assurance was lacking for EMRs privately acquired by health care facilities, while the predominant HIV care system had a well-integrated data quality system. With various programmes implementing record identification models tailored to their program objectives, it makes it difficult to pool data belonging to an individual in different databases [23]. This results in fragmented datasets, limiting the potential uses of digital data and sustainability [56].

We found interoperability efforts in Kenya affected by the wide range of actors with different EMR systems and limited coordination; differences in technology; inadequate government leadership and resource allocation to health data systems; an absence of standardisation of processes and technologies for interoperability; and concerns around data quality. Even though the MOH has made efforts through the introduction of interoperability guidelines and, recently, the digital health act [14,57], Kenya is yet to achieve a fully integrated digital healthcare system. Homa Bay, as an exemplar county, demonstrates the high EMR coverage in Kenya dominated by an HIV care platform and the existence of other EMRs in use that do not speak to each other. While we foresee a promising future of integrated digital health platforms, a lot of data already generated for healthcare could be used to answer public health research questions [58,59]. Effective data governance frameworks are essential for facilitating data sharing and integration, particularly in today’s healthcare landscape where artificial intelligence is becoming increasingly prevalent [60,61].

One of the approaches to utilise EMR routine care data is through record linkage [62]. Record linkage is an approach to link patients’ data across platforms using person-centric identifiers, especially when there is a lack of interoperability. As evidenced by our findings, none of the active EMRs had a common shared unique record identifier other than person identifiers. Record linkage can be applied to these EMR databases to generate a robust database for research studies [63,64]. However, the recent enactment of data protection guidelines in many Sub-Saharan African countries restricts person-centric identifiable health data across different platforms and institutions [65]. As such, a strong legal and regulatory basis to protect privacy and mitigate potential cybersecurity threats is required as an enabler to EMR data utilisations [60,66].

The devolved Kenyan system of government means counties control health budgets, deal directly with vendors, and decide on preferred EMR systems based on local needs and partnerships [67,68]. This local approach can allow for local oversight of partners, buy-in from county managers and tailored local solutions based on local data and problems. On the other hand, it fosters politically driven, rather than technically driven, decision-making and fragmented EMR systems [69]. The national government has been piloting digital health systems suitable for national rollout for non-HIV care, developing guidelines and supporting counties. However, varying political agendas influence digital health strategy every time there is a leadership transition [70]. Multiple not-interoperable EMRs derail efforts to maximise the impact of digital health data, especially when there is a lack of coordination of data sharing and frameworks to allow the use of routine care data in a safe, equitable manner [71]. A stable technological infrastructure to support data collection, synchronisation, storage, analysis, and visualisation across interoperable EMR systems is essential to any sustainable national system [72]. All these require financial support, both through the government and implementing partners.

**Table:** 

Recommendations
The government must take a central leadership in driving EMR implementation and defining the scope and purpose to cover the general population. Direct partners to support universally compatible EMR platforms and drive the interoperability of existing platforms. Implement a sustainable patient record identification mechanism to allow seamless data sharing amongst platforms and promote inter and intra-facility longitudinal patient follow-up. Develop legal and operating guidelines for routine healthcare data utilisation, sharing, and governance. Provide a secure, central, safe platform for data access for innovation and research for individual and public health care. Develop a data quality assurance protocol to guide EMR use and routine data quality verification while building the capacity of healthcare workers.

The study’s limitations should be considered while interpreting the results. The findings of this study are limited to one Kenyan county and may not represent the EMR situation in Kenya or other Kenyan counties. Sampling criteria were nested in two-parent studies, which limited the scope of facilities surveyed, and no views were sought for facilities without an EMR, limiting generalisability. Future research could use a defined sampling framework to cover facilities with ongoing research studies and those without. The findings are heavily skewed towards the utility of the predominant HIV care platform, and it is hard to get an alternative perspective of the situation of a multipurpose EMR. A future study could conduct a comparative survey for facilities using a non-HIV EMR. While we identified non-EMR digital health technologies, their adoption was markedly lower, and they were frequently used alongside existing EMR systems, limiting the extent to which independent conclusions could be drawn about their impact and integration.

## Conclusions

This study described the use of EMR systems in healthcare facilities while highlighting the opportunities and limitations of their use in public health decision-making or research. The study found nine different active EMRs with large coverage dominated by an HIV care management system. EMR utilisation was challenged by the lack of interoperability, unique patient identifiers, internet connectivity, non-coordinated data quality assurance processes, and dual data documentation. These challenges negatively impact the potential of data collected on a routine care basis for decision-making. The exponential data growth over time from multiple EMRs is evident. This study recommends potential strategies such as the development of a routine care data governance framework, implementation of a universal unique patient identifier, coordinated data quality efforts, directing partners to support fit all-digital system, driving interoperability of existing systems, application of record linkage methods to pool together data collected over time retrospectively, and assessment of existing digital health data for their suitability to inform research studies and ultimately for decision making.

## Supporting information

S1 Table EMR utilisation characteristics. The EMR use characteristics include challenges, EMR users, EMR devices, EMR user friendliness, data quality assurance, data reporting and review, data capture consenting and guidelines.
(PDF)
