# Supplementary material for: Missed opportunities for digital health data use in healthcare decision-making: A cross-sectional digital health landscape assessment in Homa Bay county, Kenya
Source: PLOS Digit Health. 2025 Jun 13;4(6):e0000870. doi: 10.1371/journal.pdig.0000870 (PMC12165431; doi:10.1371/journal.pdig.0000870)
Supplement: S1 Table — The EMR use characteristics include challenges, EMR users, EMR devices, EMR user friendliness, data quality assurance, data reporting and review, data capture consenting and guidelines. (PDF) [file pdig.0000870.s001.pdf]

EMR utilisation characteristics.

Characteristic	N	**Overall**, N = 112[1]	**Level 2**, N = 42[1]	**Level 3**, N = 46[1]	**Level 4 and 5**, N = 24[1]
**EMR friendliness**	87				
Agree		38 (44%)	16 (46%)	14 (47%)	8 (36%)
Disagree		7 (8.0%)	2 (5.7%)	1 (3.3%)	4 (18%)
Neutral		15 (17%)	8 (23%)	5 (17%)	2 (9.1%)
Strongly agree		23 (26%)	8 (23%)	9 (30%)	6 (27%)
Strongly disagree		4 (4.6%)	1 (2.9%)	1 (3.3%)	2 (9.1%)
Unknown		25	7	16	2
**Devices for accessing EMR**					
**Tablet**	111				
Yes		84 (76%)	33 (79%)	30 (67%)	21 (88%)
No		27 (24%)	9 (21%)	15 (33%)	3 (13%)
Unknown		1	0	1	0
**Desktop or a computer**	111				
Yes		14 (13%)	4 (9.5%)	0 (0%)	10 (42%)
No		97 (87%)	38 (90%)	45 (100%)	14 (58%)
Unknown		1	0	1	0
**Laptop**	111				
Yes		3 (2.7%)	0 (0%)	1 (2.2%)	2 (8.3%)
No		108 (97%)	42 (100%)	44 (98%)	22 (92%)
Unknown		1	0	1	0
**Mobile phone**	111				
Yes		18 (16%)	8 (19%)	5 (11%)	5 (21%)
No		93 (84%)	34 (81%)	40 (89%)	19 (79%)
Unknown		1	0	1	0
**EMR users**					
**Facility in charge**	91				
Yes		11 (12%)	4 (13%)	4 (10%)	3 (16%)
No		80 (88%)	28 (88%)	36 (90%)	16 (84%)
Unknown		21	10	6	5
**Health records information officer**	91				
Yes		22 (24%)	7 (22%)	5 (13%)	10 (53%)
No		69 (76%)	25 (78%)	35 (88%)	9 (47%)
Unknown		21	10	6	5

EMR utilisation characteristics.

Characteristic	N	**Overall**, N = 112[1]	**Level 2**, N = 42[1]	**Level 3**, N = 46[1]	**Level 4 and 5**, N = 24[1]
**Nurse**	91				
Yes		49 (54%)	17 (53%)	18 (45%)	14 (74%)
No		42 (46%)	15 (47%)	22 (55%)	5 (26%)
Unknown		21	10	6	5
**Clinician**	91				
Yes		24 (26%)	7 (22%)	10 (25%)	7 (37%)
No		67 (74%)	25 (78%)	30 (75%)	12 (63%)
Unknown		21	10	6	5
**Medical doctor**	91				
Yes		1 (1.1%)	1 (3.1%)	0 (0%)	0 (0%)
No		90 (99%)	31 (97%)	40 (100%)	19 (100%)
Unknown		21	10	6	5
**Volunteer**	91				
Yes		1 (1.1%)	0 (0%)	0 (0%)	1 (5.3%)
No		90 (99%)	32 (100%)	40 (100%)	18 (95%)
Unknown		21	10	6	5
**Interns**	91				
No		91 (100%)	32 (100%)	40 (100%)	19 (100%)
Unknown		21	10	6	5
**Other users** (HTS counsellor, peer educator, triage officers, peer mentor, HTS provider, lab technologist and CCC nurse or clinician).	91				
Yes		12 (13%)	2 (6.3%)	6 (15%)	4 (21%)
No		79 (87%)	30 (94%)	34 (85%)	15 (79%)
Unknown		21	10	6	5
**EMR record identification**	110				
Both (Manual & computer generated)		20 (18%)	7 (17%)	5 (11%)	8 (33%)
Computer generated		3 (2.7%)	2 (4.8%)	0 (0%)	1 (4.2%)
Manual		87 (79%)	33 (79%)	39 (89%)	15 (63%)
Unknown		2	0	2	0
**EMR consenting**	88				
Don't know		4 (4.5%)	1 (2.8%)	2 (6.7%)	1 (4.5%)
No		31 (35%)	13 (36%)	9 (30%)	9 (41%)
Yes		53 (60%)	22 (61%)	19 (63%)	12 (55%)

EMR utilisation characteristics.

Characteristic	N	Overall, N = 112[1]	Level 2, N = 42[1]	Level 3, N = 46[1]	Level 4 and 5, N = 24[1]
Unknown		24	6	16	2
**EMR confidentiality guidelines available**	88				
Don't know		3 (3.4%)	2 (5.6%)	0 (0%)	1 (4.5%)
No		4 (4.5%)	1 (2.8%)	2 (6.7%)	1 (4.5%)
Yes		81 (92%)	33 (92%)	28 (93%)	20 (91%)
Unknown		24	6	16	2
**Data reporting**	91				
Automatic system generated - dashboard		5 (5.5%)	2 (6.3%)	1 (2.5%)	2 (11%)
Both		48 (53%)	19 (59%)	17 (43%)	12 (63%)
Manual compilation		38 (42%)	11 (34%)	22 (55%)	5 (26%)
Unknown		21	10	6	5
**EMR data review**	91	82 (90%)	31 (97%)	34 (85%)	17 (89%)
Unknown		21	10	6	5
**EMR data reporting frequency**	91				
Monthly		79 (87%)	26 (81%)	37 (93%)	16 (84%)
On Demand		6 (6.6%)	3 (9.4%)	1 (2.5%)	2 (11%)
Weekly		6 (6.6%)	3 (9.4%)	2 (5.0%)	1 (5.3%)
Unknown		21	10	6	5
**Actions after report generation**					
**Data upload to DHIS**	91				
Yes		38 (42%)	14 (44%)	10 (25%)	14 (74%)
No		53 (58%)	18 (56%)	30 (75%)	5 (26%)
Unknown		21	10	6	5
**Uploaded to EMR**	91				
Yes		20 (22%)	4 (13%)	8 (20%)	8 (42%)
No		71 (78%)	28 (88%)	32 (80%)	11 (58%)
Unknown		21	10	6	5
**Sent to national MOH**	91				
Yes		15 (16%)	4 (13%)	5 (13%)	6 (32%)
No		76 (84%)	28 (88%)	35 (88%)	13 (68%)
Unknown		21	10	6	5

EMR utilisation characteristics.

Characteristic	N	Overall, N = 112[1]	Level 2, N = 42[1]	Level 3, N = 46[1]	Level 4 and 5, N = 24[1]
**Sent to the county**	91				
Yes		40 (44%)	11 (34%)	19 (48%)	10 (53%)
No		51 (56%)	21 (66%)	21 (53%)	9 (47%)
Unknown		21	10	6	5
**For departmental use**	91				
Yes		16 (18%)	3 (9.4%)	8 (20%)	5 (26%)
No		75 (82%)	29 (91%)	32 (80%)	14 (74%)
Unknown		21	10	6	5
**Sent to community strategy focal persons**	91				
Yes		6 (6.6%)	1 (3.1%)	3 (7.5%)	2 (11%)
No		85 (93%)	31 (97%)	37 (93%)	17 (89%)
Unknown		21	10	6	5
**To the health records information officer**	91				
Yes		28 (31%)	10 (31%)	11 (28%)	7 (37%)
No		63 (69%)	22 (69%)	29 (73%)	12 (63%)
Unknown		21	10	6	5
**Other**<br>(Sub- County, local administration, implementing partners)	91				
Yes		18 (20%)	4 (13%)	9 (23%)	5 (26%)
No		73 (80%)	28 (88%)	31 (78%)	14 (74%)
Unknown		21	10	6	5
**Data quality assurance done**	21	19 (90%)	8 (100%)	6 (86%)	5 (83%)
Unknown		91	34	39	18

[1]n (%)
